# Effects of extracellular medium conductivity on cell response in the context of sub-microsecond range calcium electroporation

**DOI:** 10.1038/s41598-020-60789-7

**Published:** 2020-02-28

**Authors:** Vitalij Novickij, Nina Rembialkowska, Gediminas Staigvila, Julita Kulbacka

**Affiliations:** 1 Institute of High Magnetic Fields, Vilnius Gediminas Technical University, Vilnius, Lithuania; 2 Department of Electrical Engineering, Vilnius Gediminas Technical University, Vilnius, Lithuania; 3 Department of Molecular and Cellular Biology, Wroclaw Medical University, Wroclaw, Poland

**Keywords:** Ion transport, Cancer models

## Abstract

In the present study, we report the effects of extracellular medium conductivity on cell response in the context of sub-microsecond range (100 ns–900 ns) electroporation, calcium electroporation and cell size. The effects of 25 ns and microsecond range (100 μs) pulses were also covered. As a model, three different cancer cell lines of various size (C32, MCF-7/DX and MC38/0) were used and the results indicated different size-dependent susceptibility patterns to the treatment. The applied pulsed electric field (PEF) protocols revealed a significant decrease of cell viability when calcium electroporation was used. The dependence of calcium ion transport and finally the anticancer effect on the external medium conductivity was determined. It was shown that small differences in conductivity do not alter viability significantly, however, mostly affect the permeabilization. At the same, MC38/0 cell line was the least susceptible to calcium electroporation, while the C32 line the most. In all cases calcium electroporation was mostly dependent on the sensitivity of cells to electroporation and could not be effectively improved by the increase of CaCl_2_ concentration from 2 mM to 5 mM. Lastly, sub-microsecond PEF stimulated aquaporin-4 and VDAC1/Porin immunoreactions in all treated cells lines, which indicated that cell water balance is affected, ions exchange is increased and release of mitochondrial products is occurrent.

## Introduction

Electroporation is a phenomenon of pulsed electric field (PEF) initiated permeabilization of biological cell plasma membrane, which serves as a basepoint of many successful biomedical and biotechnological methodologies^1,2^. The mechanism of electroporation is dependent on cell polarization in PEF and transient charge accumulation on the cell membrane (known as transmembrane potential, TMP)^3,4^. When a threshold TMP is reached, the permeability of cell membrane is increased due to occurrence of transient hydrophilic pores^5^. It is believed that oxidative effects can be a separate mechanism responsible for the formation of pores during electroporation^6,7^, nevertheless, polarization of the cell is a primary trigger.

Polarization of the cell depends on the parameters of PEF, however, also on the permittivity and conductivity of both the cells and the medium^3,8^. Therefore, the effects of extracellular medium conductivity on electroporation efficiency have been focused for decades^9–12^. Absolute majority of the scientific works focus the micro-millisecond range of pulses, while a new modality of shorter (nanosecond range) pulses was introduced and is gaining popularity^13,14^. Currently, there are two contributions^10,11^ experimentally focusing the 12–102 ns range of pulses in the context of extracellular medium conductivity, while the sub-microsecond range is not covered in literature. Both papers are published by the same group Silve *et al*., however the upmost interest lies in the peculiar cell response to 12 ns pulses. According to these studies, the same DC3-F cell line indicated an opposite response to nanosecond pulses: permeabilization induced by 12-ns pulses of moderate magnitude 32 kV/cm is higher in 1.5 S/m medium^11^, while the same pulses of 142 kV/cm are more effective in 0.1 S/m medium^10^. The lack of studies in the nanosecond and sub-microsecond range prevent forming descriptive conclusions about the mechanism of the phenomena. However, it is clear that electroporation does not linearly depend on the energy delivered to the cells, therefore, treatment protocol selection becomes a non-straightforward task due to the dramatic shifts of treatment efficacy, which currently cannot be fully understood or predicted.

At the same time, calcium electroporation is a new modality of electrochemotherapy, which is sensitive to the mentioned phenomena^15–17^. Firstly, depending on the protocol the concentration of calcium may vary several-fold (i.e. from 0.5 to 5 mM)^18^, which inevitably alters the medium conductivity. Also, during *in vitro* studies the STM buffer (popular in electroporation works) is no longer applicable since calcium and phosphates precipitate^15^, limiting the methodology to buffers like 10 mM HEPES^19^, which have different conductivities. Changes in conductivity can severely alter the efficiency of electroporation and thus make the treatment planning, permeabilization prediction, comparison or consolidation of knowledge complicated.

In this work, we present experimental data on extracellular medium conductivity effects during electroporation in the submicrosecond range (100 ns–900 ns) using mouse colon carcinoma MC38/0 cell line as a model. We superposition our data with available studies^10,11^ in the nanosecond range using 25 ns pulses of 60 kV/cm. To maximize the consolidation of knowledge and to provide a reference for the described efficacies, we also cover the conventional microsecond range (100 μs × 8) pulses. Selected treatment parameters from each range were also compared for human cancer cell lines MCF-7/DX and C32 to prove that the observed effects are consistent. Lastly, we show that calcium electroporation is dependent mostly on the sensitivity of cells to electroporation and cannot be effectively compensated by the increase of calcium concentration. The effects of different pulse parameters on the functioning of water channel (aquaporin-4) and pore-forming voltage dependent anion channel (VDAC) were studied.

## Results

### Simulation of cell transmembrane potential

**Figure 1:**
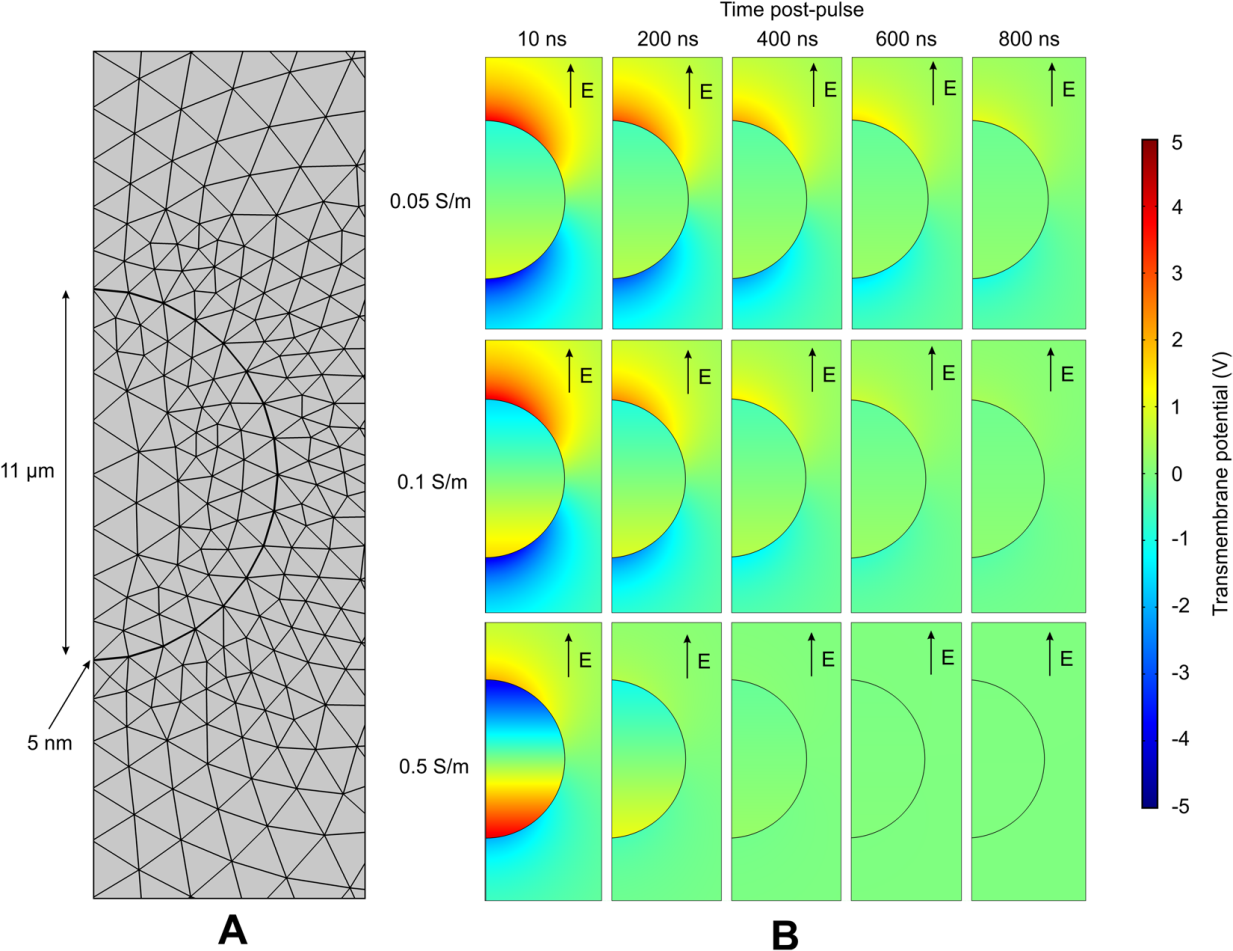
FEM simulation of cell transmembrane voltage, where (**A**) – axisymmetric mesh structure; (**B**) – post-pulse cell transmembrane potential relaxation for different conductivity mediums. Electric field direction is shown to provide the information about the pulse polarity during application of PEF.

When the physics of pore size and resealing are not introduced, the dynamics of potential relaxation does not depend on the applied pulse duration, but rather on potential amplitude and RC parameters of the system. Therefore, for analysis of potential relaxation the 8 kV/cm × 1 μs pulse was used. The results of potential relaxation in different conductivity of extracellular medium are shown in Fig. 1.

The cell was represented as an axisymmetric structure of material boundaries of different conductivity/thickness (Fig. 1A), which influenced the dynamics of cell depolarization (Refer to Fig. 1B). It can be seen in Fig. 1B that the higher was the conductivity of the extracellular medium the more effective the depolarization of the cell. Also, the polarization of the cell is altered when the conductivity of external medium is comparable or higher than the conductivity of the intracellular liquid, due to the changes of current densities.

**Figure 2:**
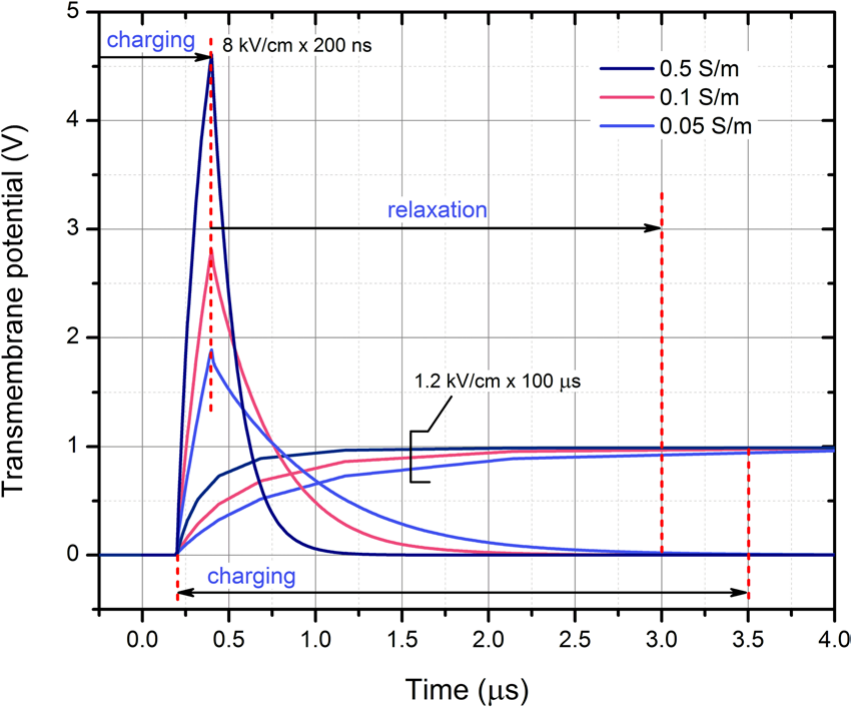
Dynamics of cell polarization and depolarization in extracellular mediums of different conductivity.

The dynamics of cell polarization and depolarization during 200 ns and 100 μs pulses are shown in Fig. 2.

As it can be seen in Fig. 2, the lowest conductivity medium (0.05 S/m) due to RC charging nature of the cell limits the charging speed of the membrane. During 100 μs pulses the transient to reach the peak potential is in the range of 3.5 μs, which in comparison to duration of the pulse is not significant. However, the situation dramatically changes in the sub-microsecond range (200 ns), when the polarization time of the cell is a lot longer compared to the pulse duration. As a result, the induced transmembrane voltage during the same pulse (8 kV/cm × 200 ns) varies dramatically between mediums of different conductivity. Even a slight change (from 0.05 to 0.1 S/m) influences the increases of transmembrane voltage up to 55% due to differences in dynamics of polarization.

### Experimental data

#### Electrotransfer in different conductivity mediums

**Figure 3:**
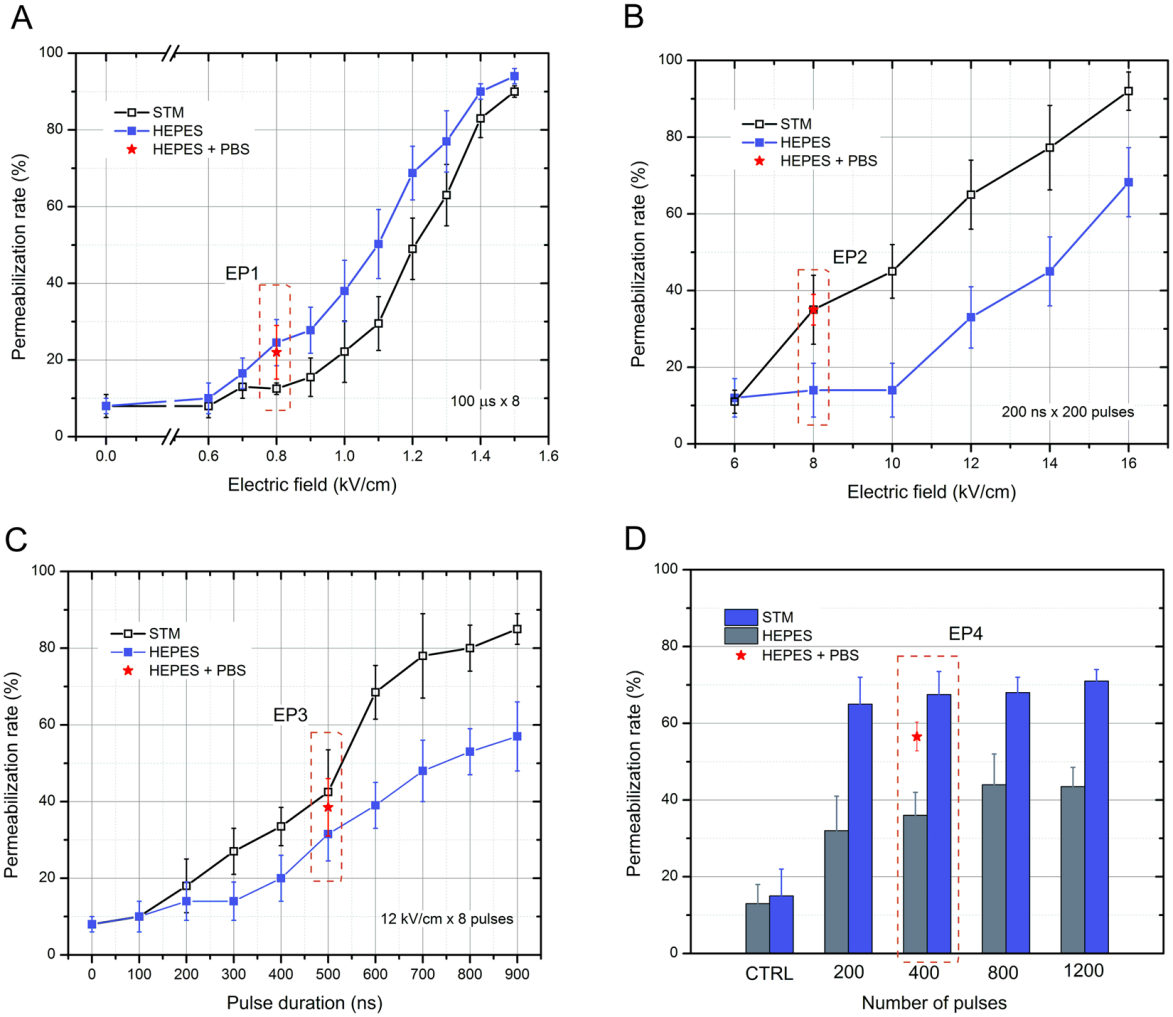
Dependence of YP uptake on applied PEF parameters and medium type in MC38 cell line, where (**A**) – conventional 100 μs × 8 protocols; (**B**) – 200 ns × 200 pulses protocols; (**C**) – 12 kV/cm × 8 pulses protocols; (**D**) – 60 kV/cm × 25 ns protocols; CTRL – untreated control; PBS – phosphate buffered saline.

The efficiency of electrotransfer for MC38/0 was evaluated in two buffers: STM – standard for *in vitro* electroporation experiments and HEPES – typical for *in vitro* calcium electroporation studies. Taken that the only difference was the buffer, the resultant conductivities were 0.1 S/m and 0.05 S/m, respectively. Parametric analysis of PEF influence on the uptake of YO-PRO-1 (YP) was performed. The results are summarized in Fig. 3. In order to establish that the changes in uptake between STM and HEPES were mainly influenced by the extracellular medium conductivity, from each range of parameters (Fig. 3A–D) a protocol was selected (EP1–EP4) to test the uptake when HEPES is mixed with highly conductive phosphate buffered saline (PBS) to a resultant conductivity of the final solution of 0.1 S/m (identical to STM based medium).

As it can be seen in Fig. 3A, for the microsecond range (100 μs × 8), lower conductivity of the medium influenced higher permeabilization rate in the whole range of PEF amplitudes (0.6–1.5 kV/cm). Matching of the HEPES-based medium conductivity to STM resulted in lower permeabilization (compared to HEPES), however the difference was not statistically significant (P > 0.05).

Further, the 200 ns × 200 pulsing sequences were used in the 6–16 kV/cm range (Fig. 3B). The differences between HEPES and STM were more profound compared to the microsecond range protocols. However, the lower conductivity buffer (HEPES) resulted in lower permeabilization efficiency compared to the higher conductivity STM, which was also predicted by the FEM simulation (Refer to Fig. 2). Matching of the HEPES based solution conductivity to the STM resulted in identical response as in STM, which is in agreement with RC model of the cell.

The same methodology was applied to test the phenomena in 100–900 ns range and a fixed amplitude PEF was used (12 kV/cm). The results are summarized in Fig. 3C. It can be seen, that the tendency is consistent in the sub-microsecond range – lower conductivity medium negatively influences the permeabilization efficiency. Matching/increasing of the conductivity (HEPES+PBS) improves the efficiency of YP uptake.

Lastly, we performed the series of experiments in the nanosecond range (60 kV/cm × 25 ns), which were also consistent with the RC model of the cell (Fig. 3D). However, we were not able to observe a significant (P < 0.05) incremental effect in permeabilization with increase of the number of the pulses from 200 to 1200.

#### Susceptibilities of human cancer cell lines to electroporation

**Figure 4:**
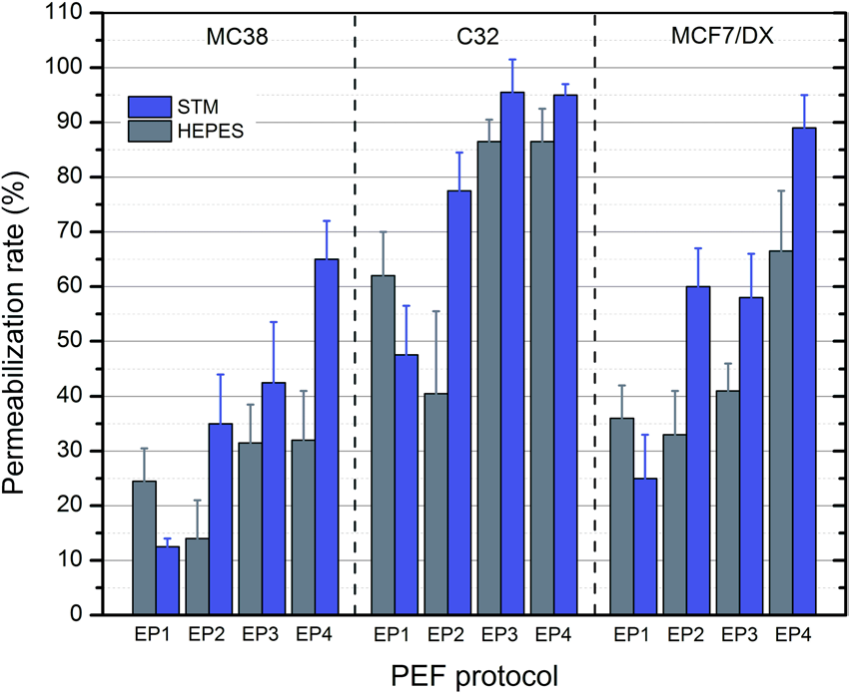
Dependence of YP uptake with selected PEF protocols between different cell lines, where EP1–0.8 kV/cm × 100 μs × 8; EP2–8 kV/cm × 200 ns × 200; EP3–12 kV/cm × 500 ns × 8; EP4–60 kV/cm × 25 ns × 400.

In order to establish that the observed effects of extracellular buffer conductivity are not cell specific, we have tested the selected protocols (EP1–EP4) on human skin melanoma (C32) and breast cancer (MCF7/DX) cell lines. The results are presented in Fig. 4.

As it can be seen in Fig. 4, different cells lines feature different susceptibility to electroporation, however, the tendency of the response in the context of extracellular medium conductivity is the same. In microsecond range (EP1), all three cell lines showed an increase in uptake of YP when a lower conductivity buffer was used (HEPES). Also, the skin amelanotic melanoma cell line was the most susceptible to the treatment followed by drug resistant breast cancer cells. The colon cancer (MC38/0) cell line was the least susceptible to PEF and the result was consistent in the whole range of parameters (EP1–EP4).

#### Metabolic activity of cells after calcium electroporation

**Figure 5:**
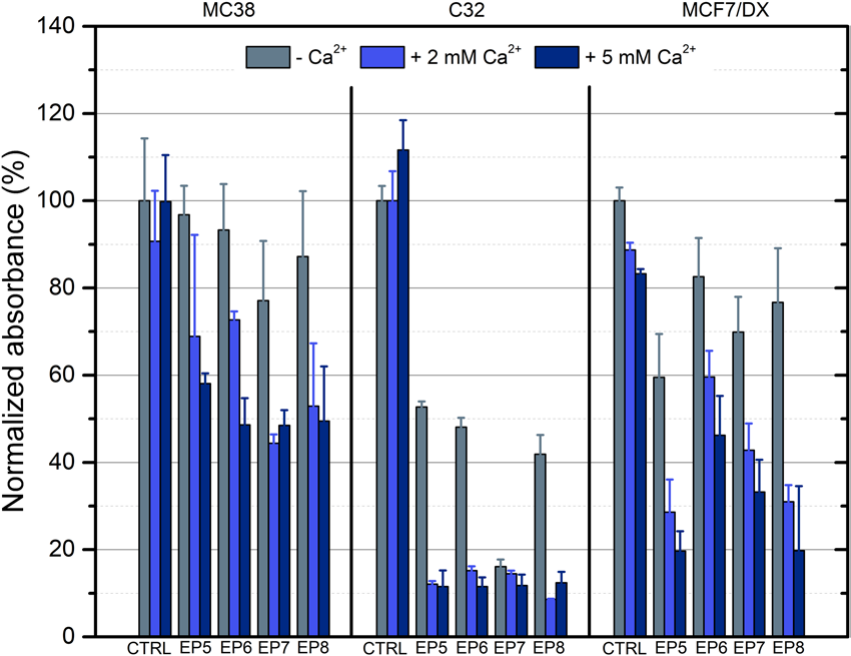
Viability of different cell lines evaluated based on MTT assay after calcium electroporation, where EP5–1.2 kV/cm × 100 μs × 8; EP6–12 kV/cm × 200 ns × 200; EP7–12 kV/cm × 800 ns × 8; EP8–60 kV/cm × 25 ns × 1200.

In order to achieve high efficiency of calcium electroporation, high permeabilization rate of the cells must be ensured to maximize the electrotransfer. Therefore, for viability evaluation experiments (based on MTT assay) the energy of (EP1–EP4) was increased to ensure saturated permeabilization (EP5–EP8). Also, two concentrations of calcium were used (2 mM and 5 mM). All the experiments were performed in HEPES buffer. The 2 mM concentration was selected based on the available knowledge on calcium electroporation. It is known that a threshold in concentration exists when the calcium electrotransfer starts to be effective, while 2 mM is an optimal dose across many cell lines^20^. The resultant conductivity of the medium during the 2 mM Ca^2+^ electroporation procedure was 0.08 S/m. The 5 mM concentration (medium conductivity of 0.1 S/m) was selected to test if there is any treatment efficacy dependence on calcium concentration in the sub-microsecond range, which was not covered in literature previously. The results are summarized in Fig. 5.

As it can be seen in Fig. 5, the metabolic activity data are in agreement with permeabilization experiments in terms of different susceptibility of cells to pulsed electric field. The C32 line was the most susceptible to treatment, followed by MCF7/DX. The MC38/0 line showed the weakest response to the treatment. The differences in efficacy between calcium concentrations were statistically non-significant in absolute majority of experimental instances independently on the cell line, which implies that the efficacy of calcium electroporation is mostly dependent on the applied parameters of PEF/cell susceptibility and cannot be compensated by the increase of the calcium dose.

Taking into account the difference in electroporation efficacy between different conductivity buffers, we also expected to observe a reflection of the observed phenomena in the viability data. However, it was not the case. Independently on the applied protocol, the differences in metabolic activity in the context of extracellular medium conductivity were not statistically significant.

#### Aquaporin-4 and VDAC1/Porin immunostaining

The expression of two membrane proteins: aquaporin-4 (AQ-4), which forms a water-specific channel and VDAC1/Porin (a mitochondrial channel involved in cell volume regulation and apoptosis) were further analyzed in the study.

**Figure 6:**
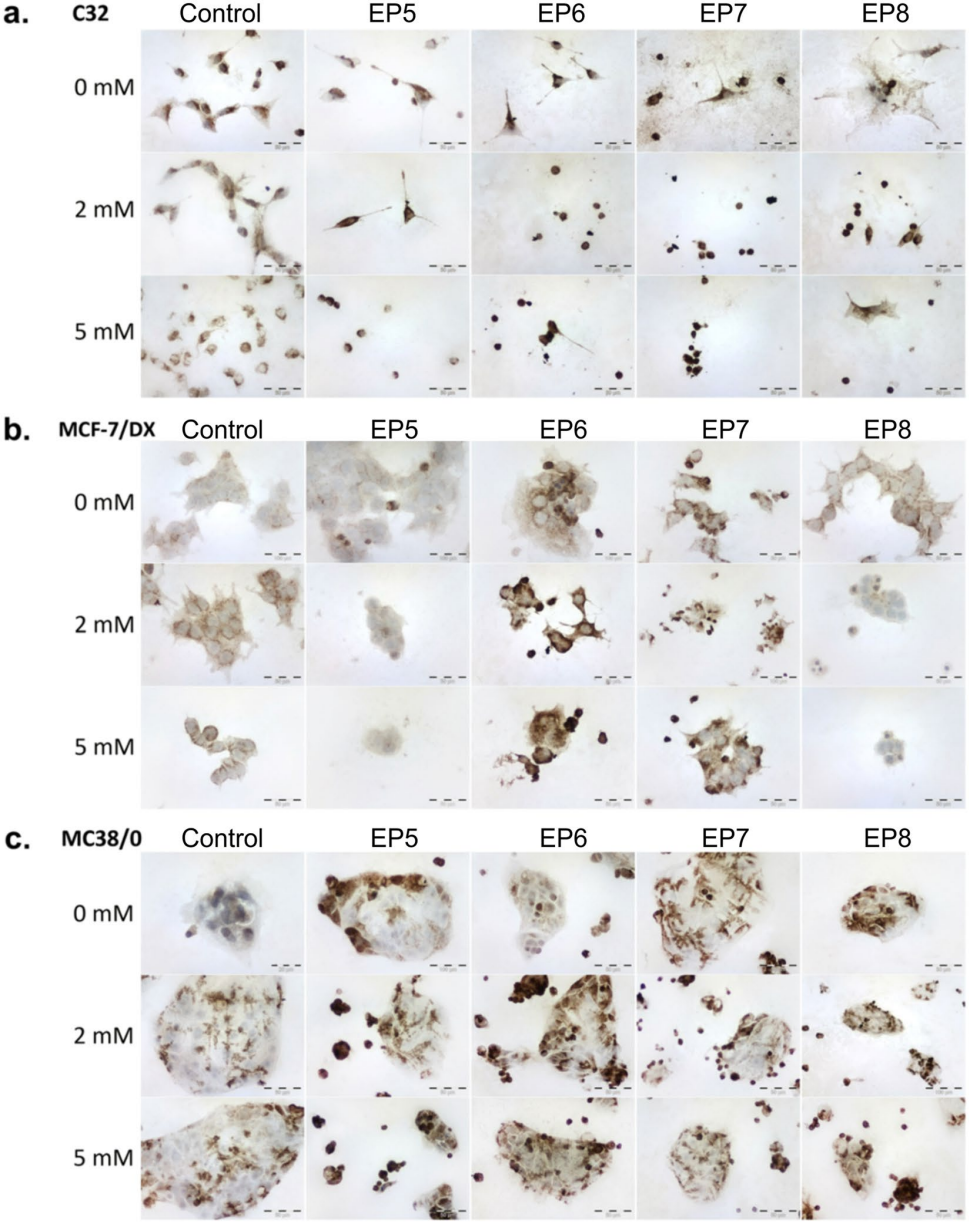
Immunoassayed reaction with anti-aquaporin-4 antibody detected in (**a**) amelanotic human melanoma cells (C32); (**b**) human resistant breast adenocarcinoma cells (MCF-7/DX) and (**c**) murine colon adenocarcinoma cell (MC38/0), where EP5–1.2 kV/cm × 100 μs × 8; EP6–12 kV/cm × 200 ns × 200; EP7–12 kV/cm × 800 ns × 8; EP8–60 kV/cm × 25 ns × 1200.

In case of melanoma (C32) the number of cells was reduced and the morphology was altered after electroporation and in particular with calcium ions (Table 1 and Fig. 6a). We have observed a significant decrease of cell volume due to cell shrinkage and loss of adhesion by filopodia. However, the levels of AQ-4 were the same as in the control samples without PEF treatment.

**Table 1:** Positive grading quantification of immunocytochemical staining of Aquaporin-4 expression in the three different cell lines: C32 – human amelanotic melanoma; MCF-7/DOX – human breast adenocarcinoma cells resistant to doxorubicin; MC38/0 – murine colon adenocarcinoma.

CaCl_2_	Control	EP5	EP6	EP7	EP8
**C32**					
0 mM	100%, ++	95% ++/+++	100%, +++	100%, +++	100%, +++
2 mM	100%, ++	100%, +++	100%, +++ (strong cell shrinkage)	100%, +++ (strong cell shrinkage)	100%, +++
5 mM	98%, ++/+++	97%, +++	100%, +++	100%, +++ (strong cell shrinkage)	100%, ++/+++
**MCF-7/DOX**					
0 mM	35%, −/+	45% ++	57%, ++/+++	68% +++	55% ++
2 mM	98% ++	36%, ++	97%, +++	74%, +++ (reduced cell size)	45%, + (reduced cell size and number)
5 mM	97% +/++	10%, +/++	100%, +++	85%, ++/+++	42%, + (reduced cell number)
**MC38/0**					
0 mM	23%; +	32%, ++/+++	24%, ++	46%, ++/+++	32%, ++/+++
2 mM	61%; ++	42%, ++/+++	77%, ++/+++	53%, ++/+++	51%, +++
5 mM	78%; ++	91%, +++	81%, ++/+++	62%, ++/+++	79%, +++

In case of breast adenocarcinoma cells (MCF-7/DX) a slight increase of AQ-4 immunoassayed reaction when exposed to calcium ions was observed. The combination with PEF treatment triggered an increase of AQ-4 expression, i.e. EP6 (97% positive cells with 2 mM Ca^2+^). At the same time, EP4 parameter caused a slight decrease of the immune reaction, however, cell number was significantly reduced. Colon cancer cells revealed relatively low expression of AQ-4 in control samples (23%), while both calcium ions and electroporation alone increased the immunostained reaction (61% and 78%, respectively). The combined protocols (PEF + CaCl_2_) enhanced the intensity of the reaction and the percentage of expressing cells for all protocols.

**Figure 7:**
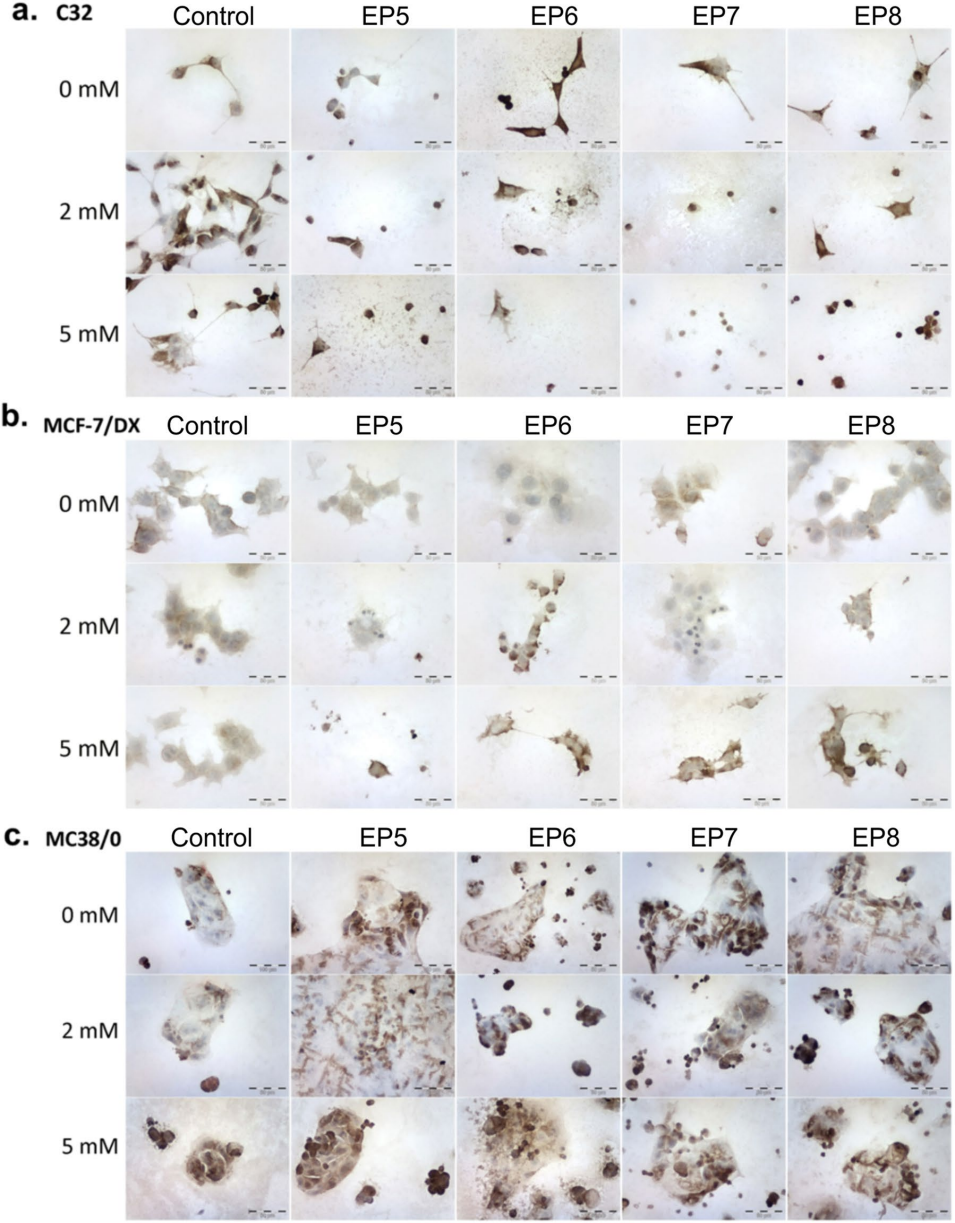
Immunoassayed reaction with anti-VDAC1 antibody detected in (**a**) amelanotic human melanoma cells (C32); (**b**) human resistant breast adenocarcinoma cells (MCF-7/DX) and (**c**) murine colon adenocarcinoma cell (MC38/0), where EP5–1.2 kV/cm × 100 μs × 8; EP6–12 kV/cm × 200 ns × 200; EP7–12 kV/cm × 800 ns × 8; EP8–60 kV/cm × 25 ns × 1200.

Various levels of expression of VDAC1-porin channel in C32, MCF-7/DX and MC38 cells were also investigated. Amelanotic melanoma and colon cancer cells showed the most intense reaction, whereas lower level of the reaction was exhibited in resistant breast cancer cells. The results of VDAC1 immunoassay are presented in Table 2 and Fig. 7. A significant increase of the immune reaction after calcium electroporation was observed (100% stained cells). The altered cell morphology, i.e. reduction of cell size due to shrinking and a reduced number of cells were detected (Fig. 7a). In case of breast cancer cells the increase of VDAC1 expression was detected for nanosecond protocols EP6, EP7, EP8, and when calcium electroporation was used (Fig. 7b). Similarly, colon cancer cells showed the highest increase of VDAC1 expression when CaEP was used (almost 100% of stained cells). Nanosecond range pulses provoked breaking up of the cells from grouped culture into smaller populations and cell shrinking (Fig. 7c).

**Table 2:** Positive grading quantification of immunocytochemical staining of VDAC1 expression in the three different cell lines: C32 – human amelanotic melanoma; MCF-7/DOX – human breast adenocarcinoma cells resistant to doxorubicin; MC38/0 – murine colon adenocarcinoma.

CaCl_2_	Control	EP5	EP6	EP7	EP8
**C32**					
0 mM	71%, ++	83%, ++	100%, +++	100%, +++	94%, ++/+++
2 mM	88%, ++/+++	99%, +++	100%, +++	100%, +++	100%, +++
5 mM	89%, ++/+++	100%, +++	100%, +++ (reduced cell number)	99%, ++/+++ (cell shrinkage)	100%, +++ (cell shrinkage)
**MCF-7/DOX**					
0 mM	65%, +/++	53%, +	15%, +	88%, ++	69%, +
2 mM	91%, ++	57%, ++	98%, ++/+++	67%, +/++	77%, ++ (cell shrinkage)
5 mM	90%, ++	96%, +++ (reduced cell number)	100%, ++/+++	100%, ++/+++	100%, +++
**MC38/0**					
0 mM	26%, ++	59%, ++/+++	75%, ++/+++	67%, +++	61%, ++
2 mM	34%, ++	86%, ++	82%, +++	78%, +++	83%, +++
5 mM	100%, +++	100%, +++	100%, +++	96%, +++	100%, +++

## Discussion

In the present study, we report the effects of extracellular medium conductivity on cell response in the context of sub-microsecond range (100–900 ns) electroporation, calcium electroporation and cell size. The results are superpositioned with intense 25 ns pulses and conventional (100 μs × 8) microsecond range treatment.

The external medium conductivity had a major impact on the permeabilization rate of the cells in all the described electroporation ranges. For sub-microsecond range pulses, the data were in perfect agreement with the RC model of the cell predicting that the lower conductivity medium will reduce the permeabilization efficiency. Basically, the RC constant is too high for the effective charging of the cell membrane, thus lower TMP is induced even due to the slight reduction of conductivity. In the microsecond range the situation is the opposite and there is a controversy with the simple RC model, however, recently it was proven that microsecond range pulses additionally induce deformation during the pulse^21^. As a result, the altered cell form-factor influences the induced TMP and thus permeabilization efficiency^12^. Our experimental data are in agreement with available experimental works^10,12,22^, therefore, taking into account the deformation factor experienced during microsecond range electroporation, the mechanism seems plausible. For nanosecond pulses (25 ns) we were not able to observe the shifts in response between the low and high conductivity buffers as it was highlighted by Silve *et al*., previously^11^. However, we have used 60 kV/cm PEF, which can be still non-sufficient to observe the effect since the shift occurs only in extremely high field according to the mentioned study. Therefore, our data indicate that in the submicrosecond range (25 ns–900 ns) with field intensities up to 60 kV/cm, the response is consistent with the conventional RC response of the cell (lower conductivity = slower membrane charge and lower permeabilization).

We have also determined different susceptibility patterns for three cells lines and the results are in agreement with the size distribution of the selected cells. The smallest MC38/0 line was least susceptible, while the biggest C32 line was the most sensitive to the treatment. According to the established electroporation theory and the Krassowska cell model^3^, the induced TMP is the lowest for MC38/0, which shows no controversy with the observed susceptibility patterns. In the context of calcium electroporation the data obtained from viability assay follows the same susceptibility pattern. Additionally, calcium electroporation increased the conductivity of the suspension and thus affected the treatment also purely through polarization. Nevertheless, small differences in conductivity do not alter viability significantly, however mostly affect permeabilization, which is useful for protocol optimization purposes. This seems promising for applications where high permeabilization with minimum loss in viability is required such as electrotransfection. It was also shown that calcium electroporation cannot be effectively improved by the increase of calcium concentration above the threshold. The result is in agreement with the study^23^ by Wasson *et al*. During study design we speculated that the ultrashort pulses will result in lower electrotransfer of calcium compared to the conventional procedures, thus increase of the concentration will allow to compensate for the loss. Partially it was true and the difference in treatment efficiency between 2 mM and 5 mM calcium concentrations was more profound in sub-microsecond range compared to the ESOPE protocol (Refer to Fig. 5), however, the differences were not statistically significant. It implies that 2 mM calcium concentration ensures almost saturated cell inactivation efficiency independently on the pulse range.

Lastly, the expression of two membrane proteins AQ-4 and VDAC1/Porin were investigated in the study to determine the influence of sub-microsecond pulses on the cell water balance, ions exchange and the release of mitochondrial products. Mitochondrial channels are responsible for regulation of mitochondrial ATP and calcium flux^24^. VDAC1/porin mitochondrial channel can control energy and cell metabolism, and as we know calcium ions can modulate its activity^24^. Thus, the processes that can affect lipid organization of cell membrane can also interfere with the action of membrane channels. In our study, the process of electroporation changed the expression of aquaporin-4 and VDAC1 channel. We suppose that electrical pulses can stimulate cell to overexpress transmembrane channels (here AQ-4 and VDAC1) and increase the flow of solute through the membrane. Thus, in the context of “electropores” additional ways of transport are created. We assume that EP can induce overexpression of transmembrane proteins and supposedly can inhibit the channel in an open state or stimulate its activity. Such a phenomenon can explain the loss of cell volume. Previously, it was shown that cell volume change for e.g. after electroporation can lead to the consequent activation of chloride and potassium channels, with simultaneous water flow via aquaporins^25^. Recently, it was also highlighted that ion channels are quite promising targets in anticancer protocols. Ion channels are responsible for numerous cellular processes and their expression varies in different types of cancers^26,27^. In our study, an increase of expression of water channels and mitochondrial VDAC1 was determined, which can significantly support the cytotoxic and anticancer effect caused by EP and CaEP. The disruption of the channel functioning is an additional factor that may cause cell death^26,27^. Molecular dynamics study involving electropermeabilization effect on transmembrane water channels (aquaporins) demonstrated a significant effect of water self-diffusion during and immediately after the pulses^28^. Further studies revealed that electric pulses can play a role in gating mechanism, hence influencing water permeability^29^. Thus, the modulation of selected channels expression and activity by electroporation seem to be a relatively good approach. However, further research is required.

To conclude, we have presented an experimental coverage of the cellular effects of PEF in the sub-microsecond range in the context of medium conductivity, calcium electroporation and cell size. We have also, provided data indicating the influence of sub-microsecond pulses on water and mitochondrial channels, which can be an additional way for molecules delivery, despite “nanopores”. The results may have application in optimization of PEF parameters for calcium electroporation and establishment of new approaches employing ion channel transport in anticancer therapy.

## Material and Methods

### Pulsed power setups

The experimental setup consisted of 3 kV, 100 ns–1 ms square wave high voltage pulse generator (VGTU, Vilnius, Lithuania)^30^ and a commercially available electroporation cuvette with 1 mm gap between electrodes (Biorad, Hercules, USA). For 25 ns pulse delivery the PPG-20 generator (FID Technology, Germany) was used. The voltage (V_C_) that was applied to the cuvette was varied in the 0.06–6 kV range, corresponding to 0.6–60 kV/cm electric field. Several groups of pulsing protocols were used: 1) 0.6–1.6 kV/cm × 100 μs × 8/1 Hz; 2) 6–16 kV/cm × 200 ns × 200/1 kHz; 3) 12 kV/cm × 100–900 ns × 8/1 Hz; and 4) 60 kV/cm × 25 ns × 200–1200/200 Hz.

**Figure 8:**
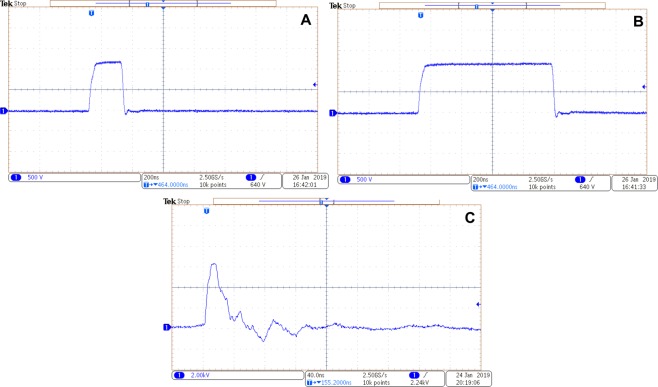
The representative waveforms of the pulses. Acquired using DPO4034 digital oscilloscope (Tektronix, Beaverton, USA), where (**A**) 200 ns pulses; (**B**) 900 ns pulse; (**C**) 25 ns pulse; the colors are inverted.

The waveforms for 2–4 groups of protocols is shown in Fig. 8. The waveform for the ESOPE range protocols (1) is not shown since it is conventional. The waveform for 2^nd^ and 3^rd^ group of PEF protocols (Fig. 8A,B) do not feature significant overshoots/transient processes. However, the 4^th^ group of pulses (Fig. 8C) has some oscillations mainly due to non-perfect impedance matching, which was a limitation of our infrastructure. The 25 ns duration was determined by the evaluation of the width of the pulse at 50% of the peak amplitude.

For C32 and MCF7/DX cell lines eight PEF protocols were used (EP1-EP8): EP1–0.8 kV/cm × 100 μs × 8; EP2–8 kV/cm × 200 ns × 200; EP3–12 kV/cm × 500 ns × 8; EP4–60 kV/cm × 25 ns x 400; EP5–1.2 kV/cm × 100 μs × 8; EP6–12 kV/cm × 200 ns × 200; EP7–12 kV/cm × 800 ns × 8; EP8–60 kV/cm × 25 ns × 1200.

### Finite element method model

The 2D axisymmetric finite element method (FEM) model of the cell in PEF was designed using COMSOL Multiphysics (COMSOL, Stockholm, Sweden). The input electrical pulse (8 kV/cm) was programmed by combining the rectangular and analytic COMSOL functions. The cell size was chosen to be 11 μm to simulate a MC38 cell line^31^. The cell membrane was defined as a thin 5 nm layer^3^ of resistive material (2.5 × 10^−7^ S/m)^32^. Intracellular medium conductivity^30^ was selected to be 0.5 S/m, while the extracellular medium conductivity was varied in 0.05–0.5 S/m range. Complete free triangular mesh consisted of 28049 domain elements and 513 boundary elements. Transmembrane potential charging was evaluated for 8 kV/cm × 200 ns and 1.2 kV/cm × 100 μs pulses.

### Cell culture

The studies were performed *in vitro* on human breast adenocarcinoma cell line doxorubicin-resistant type (MCF-7/DX), obtained from the Department of Tumor Biology, Comprehensive Cancer Center, Maria Sklodowska-Curie Memorial Institute in Gliwice (Poland), human amelanotic melanoma cells (C32) purchased in ATCC^®^, MC38 murine colon adenocarcinoma cells were adapted to *in vitro* conditions in Ludwik Hirszfeld Institute of Immunology and Experimental Therapy, Polish Academy of Sciences (Wroclaw, Poland)^33^. MCF-7/DX and C32 cells were grown in DMEM (Sigma, Poland), supplemented with 10% fetal bovine serum (Lonza BioWhittaker, Switzerland) and penicillin/streptomycin (Sigma, Poland). MC38 cells were maintained RPMI (Sigma, Poland) supplemented with 5% fetal bovine serum (FBS, Lonza BioWhittaker, Switzerland), 1% penicillin/streptomycin (Sigma, Poland), 0.5% sodium pyruvate (Sigma-Aldrich) and 50 µmol/L 2-mercaptoethanol (Sigma-Aldrich). Cell cultures were cultivated as a monolayer on a plastic flask 25 and 75 cm^2^ (Nunc, Denmark), maintained in a humidified atmosphere at 37 °C and 5% CO_2_. and detached for the experiments by trypsinization (trypsin 0.025% and EDTA 0.02% solution, Sigma, Poland). Cells were passed every 2–3 days and a day before the experiment.

### Electroporation

For electroporation cells were trypsinized and centrifuged (5 min, 1000 rpm, Centrifuge MPW-341 with stable rotor, MPW Med. Instruments, Poland). Then cells were counted and for each sample 5 × 10^5^ of cells were resuspended in STM buffer (10 mM phosphate (Chempur, Poland), 1 mM MgCl_2_ (POCH, Poland), 250 mM sucrose (POCH, Poland); pH 7.4) or HEPES buffer (10 mM, Sigma) containing 250 mM sucrose (POCH, Poland) and 1 mM MgCl_2_ in MilliQ water. For calcium electroporation, CaCl_2_ was added for a final 2 mM or 5 mM concentration. After pulse delivery, the cells were incubated for 10 min at room temperature.

### Viability assay

MTT assay was performed 24 h after the end of EP experiments to evaluate cells mitochondrial function as a viability marker. Cells were incubated with 100 μl of the MTT [3-(4,5-Dimethylthiazol-2-yl)-2,5-Diphenyltetrazolium Bromide] reagent (Sigma, Poland) at 37 °C for 1.5 h. Then, formazan crystals were dissolved with addition of 100 μl of acidic isopropanol and mixed. The absorbance was measured at 570 nm using multiwell plate reader (EnSpire Multimode Reader; Perkin Elmer, USA). The results were expressed as the percentage of mitochondrial function relative to untreated control cells. Experiments were repeated minimum three times in triplicate.

### Flow cytometry and spectrophotometry

Flow cytometry analysis was performed to evaluate electroporation efficacy through the assessment of the ability of cells to internalize impermeant dye – Yo-pro-1. Immediately before EP, YO-PRO™-1 iodide (YP-1, λ_exc_491/λ_em_509, Thermo Scientific, Poland) was added to the cell suspension. Concentration of YP-1 in the STM or HEPES buffer was 1 μM. Flow cytometric analysis was performed using CyFlow CUBE-6 flow cytometer (Sysmex, Poland). The samples were excited using the 488-nm line of the blue laser and the fluorescence of YP-1 was measured with FL-1 detector. Data were analyzed using CyView software (Sysmex).

### Immunocytochemical staining

After the EP experiment cells were seeded on 10-well slides (Thermo Scientific, USA), incubated for 24 hours, rinsed with PBS and fixed using 4% paraformaldehyde. Then immunocytochemistry was performed using the EXPOSE Mouse and Rabbit Specific HRP/DAB Detection IHC kit (Abcam, USA, ab80436). Briefly, after rinsing with PBS (3 × 5 min), peroxidase activity was blocked by 30 min incubation with 1% H_2_O_2_ and samples were permeabilized by incubation with 1% Triton X-100 (Sigma, Poland) in PBS (LabEmpire, Poland). Then the cells were incubated with selected antibodies for overnight at 4 °C. The following primary antibodies (diluted 1:200, purchased from Abcam, USA) were used: anti-Aquaporin 4 antibody [4/18] – a mouse monoclonal IgG (ab9512, Abcam), anti-VDAC1/Porin antibody – a rabbit polyclonal IgG (ab34726, Abcam). Then cells were incubated with the secondary antibody conjugated with horseradish peroxidase (HRP). Next, samples were incubated with a diaminobenzidine-H_2_O_2_ mixture in order to visualize the HRP label to visualize the peroxidase label and counterstained with hematoxylin (Roth, Poland) for 3 min. After dehydration in ethanol gradient (Chempur, Poland) and xylene (Chempur, Poland) microscopic slides were covered using DPX (Aqua-Med Zpam-Kolasa, Poland). The samples were examined using an upright microscope (Olympus BX53, Poland). Number of stained cell was determined by counting 100 cells in 3 randomly selected fields. The intensity of immunohistochemical staining was evaluated as (−) negative (no reaction), (+) weak, (++) moderate, and (+++) strong.

### Statistical analysis

One-way analysis of variance (ANOVA; P < 0.05) was used to compare different treatments. Tukey HSD multiple comparison test for evaluation of the difference was used when ANOVA indicated a statistically significant result (P < 0.05 was considered statistically significant). The data was post-processed in OriginPro software (OriginLab, Northampton, MA, USA). All experiments have been performed at least in triplicate and the treatment efficiency was expressed as mean ± standard deviation.

## Data Availability

The data are available from VN on request.

## References

[1] Rems L, Miklavčič D (2016). Tutorial: Electroporation of cells in complex materials and tissue. J. Appl. Phys..

[2] Tsong TYY (1991). Electroporation of cell membranes. Biophys. J..

[3] Krassowska W, Filev PD (2007). Modeling Electroporation in a Single Cell. Biophys. J..

[4] Marszalek P, Liu DS, Tsong TY (1990). Schwan equation and transmembrane potential induced by alternating electric field. Biophys. J..

[5] Vernier, P. T. *et al*. Nanoelectropulse-driven membrane perturbation and small molecule permeabilization. *BMC Cell Biol*. **7**, 10.1186/1471-2121-7-37 (2006). 10.1186/1471-2121-7-37 PMC1624827 17052354

[6] Pakhomova ON (2012). Oxidative effects of nanosecond pulsed electric field exposure in cells and cell-free media. Arch. Biochem. Biophys..

[7] Rems L, Viano M, Kasimova MA, Miklavčič D, Tarek M (2019). The contribution of lipid peroxidation to membrane permeability in electropermeabilization: A molecular dynamics study. Bioelectrochemistry.

[8] Pethig R (2010). Dielectrophoresis: Status of the theory, technology, and applications. Biomicrofluidics.

[9] Pucihar G, Kotnik T, Kandušer M, Miklavčič D (2001). The influence of medium conductivity on electropermeabilization and survival of cells *in vitro*. Bioelectrochemistry.

[10] Silve, A., Leray, I., Poignard, C. & Mir, L. M. Impact of external medium conductivity on cell membrane electropermeabilization by microsecond and nanosecond electric pulses. *Sci. Rep*. **6**, 10.1038/srep19957 (2016). 10.1038/srep19957 PMC4734290 26829153

[11] Silve A, Leray I, Leguèbe M, Poignard C, Mir LM (2015). Cell membrane permeabilization by 12-ns electric pulses: Not a purely dielectric, but a charge-dependent phenomenon. Bioelectrochemistry.

[12] Ruzgys, P., Jakutavičiūtė, M., Šatkauskienė, I., Čepurnienė, K. & Šatkauskas, S. Effect of electroporation medium conductivity on exogenous molecule transfer to cells *in vitro*. *Sci. Rep*. **9**, 10.1038/s41598-018-38287-8 (2019). 10.1038/s41598-018-38287-8 PMC6363740 30723286

[13] Chopinet L, Rols MP (2015). Nanosecond electric pulses: A mini-review of the present state of the art. Bioelectrochemistry.

[14] Weaver JC, Smith KC, Esser AT, Son RS, Gowrishankar TR (2012). A brief overview of electroporation pulse strength-duration space: A region where additional intracellular effects are expected. Bioelectrochemistry.

[15] Frandsen SK, Gissel H, Hojman P, Eriksen J, Gehl J (2014). Calcium electroporation in three cell lines: A comparison of bleomycin and calcium, calcium compounds, and pulsing conditions. Biochim. Biophys. Acta - Gen. Subj..

[16] Frandsen Stine Krog, Gibot Laure, Madi Moinecha, Gehl Julie, Rols Marie-Pierre (2015). Calcium Electroporation: Evidence for Differential Effects in Normal and Malignant Cell Lines, Evaluated in a 3D Spheroid Model. PLOS ONE.

[17] Zielichowska A, Daczewska M, Saczko J, Michel O, Kulbacka J (2016). Applications of calcium electroporation to effective apoptosis induction in fibrosarcoma cells and stimulation of normal muscle cells. Bioelectrochemistry.

[18] Szewczyk A (2018). Calcium electroporation for treatment of sarcoma in preclinical studies. Oncotarget.

[19] Romeo Stefania, Sannino Anna, Scarfì Maria Rosaria, Vernier P. Thomas, Cadossi Ruggero, Gehl Julie, Zeni Olga (2018). ESOPE-Equivalent Pulsing Protocols for Calcium Electroporation: An In Vitro Optimization Study on 2 Cancer Cell Models. Technology in Cancer Research & Treatment.

[20] Frandsen Stine Krog, Gehl Julie (2017). Effect of calcium electroporation in combination with metformin in vivo and correlation between viability and intracellular ATP level after calcium electroporation in vitro. PLOS ONE.

[21] Shamoon D., Dermol-Černe J., Rems L., Reberšek M., Kotnik T., Lasquellec S., Brosseau C., Miklavčič D. (2019). Assessing the electro-deformation and electro-poration of biological cells using a three-dimensional finite element model. Applied Physics Letters.

[22] Pavlin M (2005). Effect of cell electroporation on the conductivity of a cell suspension. Biophys J..

[23] Wasson Elisa M., Alinezhadbalalami Nastaran, Brock Rebecca M., Allen Irving C., Verbridge Scott S., Davalos Rafael V. (2020). Understanding the role of calcium-mediated cell death in high-frequency irreversible electroporation. Bioelectrochemistry.

[24] Tomasello Marianna F., Guarino Francesca, Reina Simona, Messina Angela, De Pinto Vito (2013). The Voltage-Dependent Anion Selective Channel 1 (VDAC1) Topography in the Mitochondrial Outer Membrane as Detected in Intact Cell. PLoS ONE.

[25] Carr, L. *et al*. Calcium-independent disruption of microtubule dynamics by nanosecond pulsed electric fields in U87 human glioblastoma cells. *Sci. Rep*. **7**, 10.1038/srep41267 (2017). 10.1038/srep41267 PMC5259788 28117459

[26] Shoshan-Barmatz V, Ben-Hail D, Admoni L, Krelin Y, Tripathi SS (2015). The mitochondrial voltage-dependent anion channel 1 in tumor cells. *Biochimica et Biophysica Acta*. Biomembranes.

[27] Xia, J. *et al*. Ion channels or aquaporins as novel molecular targets in gastric cancer. *Molecular Cancer* **16**, 10.1186/s12943-017-0622-y (2017). 10.1186/s12943-017-0622-y PMC5338097 28264681

[28] Reale Riccardo, English Niall J., Garate José-Antonio, Marracino Paolo, Liberti Micaela, Apollonio Francesca (2013). Human aquaporin 4 gating dynamics under and after nanosecond-scale static and alternating electric-field impulses: A molecular dynamics study of field effects and relaxation. The Journal of Chemical Physics.

[29] Marracino Paolo, Liberti Micaela, Trapani Erika, Burnham Christian, Avena Massimiliano, Garate José-Antonio, Apollonio Francesca, English Niall (2016). Human Aquaporin 4 Gating Dynamics under Perpendicularly-Oriented Electric-Field Impulses: A Molecular Dynamics Study. International Journal of Molecular Sciences.

[30] Novickij V (2016). High-frequency submicrosecond electroporator. Biotechnol. Biotechnol. Equip..

[31] Shashni B (2018). Size-based Differentiation of Cancer and Normal Cells by a Particle Size Analyzer Assisted by a Cell-recognition PC Software. Biol. Pharm. Bull..

[32] Ivorra A, Villemejane J, Mir LM (2010). Electrical modeling of the influence of medium conductivity on electroporation. Phys. Chem. Chem. Phys..

[33] Weeber EJ (2002). The role of mitochondrial porins and the permeability transition pore in learning and synaptic plasticity. J. Biol. Chem..

